# Effects of Intravenous Iron Replacement Therapy on Cardiovascular Outcomes in Patients with Heart Failure: A Systematic Review and Meta-Analysis

**DOI:** 10.3390/jcdd10030116

**Published:** 2023-03-11

**Authors:** Johannes Reinhold, Vyas Burra, Natasha Corballis, Vasiliki Tsampasian, Gareth Matthews, Charikleia Papadopoulou, Vassilios S. Vassiliou

**Affiliations:** 1 Norwich Medical School, University of East Anglia (UEA), Norwich Research Park, Norwich NR4 7TJ, UK; 2 Department of Cardiology, Norfolk and Norwich University Hospital, Colney Lane, Norwich NR4 7UY, UK; 3 Medical School, King’s College London, Strand, London WC2R 2LS, UK; 4 Department of Biochemistry, University of Cambridge, 80 Tennis Court Road, Cambridge CB2 1GA, UK; 5 Royal Papworth Hospital, Papworth Rd, Trumpington, Cambridge CB2 0AY, UK

**Keywords:** heart failure, iron deficiency, iron replacement, meta-analysis

## Abstract

(1) Background: Iron deficiency (ID) is an important adverse prognostic marker in patients with heart failure (HF); however, it is unclear whether intravenous iron replacement reduces cardiovascular mortality in this patient group. Here, we estimate the effect of intravenous iron replacement therapy on hard clinical outcomes following the publication of IRONMAN, the largest trial in this field. (2) Methods: In this systematic review and meta-analysis, prospectively registered with PROSPERO and reported according to PRISMA guidelines, we searched PubMed and Embase for randomized controlled trials investigating intravenous iron replacement in patients with HF and co-existing ID. The primary outcome was cardiovascular mortality and secondary outcomes were all-cause mortality, hospitalizations for HF and a combination of the primary outcome and hospitalizations for HF. (3) Results: A total of 1671 items were identified and after removal of duplicates we screened titles and abstracts of 1202 records. Some 31 studies were identified for full-text review and 12 studies were included in the final review. The odds ratio (OR) for cardiovascular death using a random effects model was 0.85 (95% CI 0.69 to 1.04) and for all-cause mortality it was 0.83 (95% CI 0.59 to 1.15). There was a significant reduction in hospitalizations for HF (OR 0.49, 95% CI 0.35 to 0.69) and the combination of hospitalizations for HF and cardiovascular death (OR 0.65, 95% CI 0.5 to 0.85). (4) Conclusions: This review supports the use of IV iron replacement reducing hospitalization rates for HF, however more research is required to determine the effect on cardiovascular mortality and to identify the patient population most likely to benefit.

## 1. Introduction

Iron is an essential micronutrient required for fundamental cellular processes including regulation of proteins, lipids and ribonucleic acids supporting oxygen transport and storage, mitochondrial function, muscle metabolism as well as immune and neural function [1,2,3,4,5]. The most frequently used definition of iron deficiency (ID) in patients with cardiovascular disease is ferritin < 100 µg/L or ferritin 100–299 µg/L and transferrin saturation (TSAT) < 20% [6,7,8], however recent studies suggested that relying on ferritin for diagnosis of iron deficiency may be suboptimal [9,10].

ID is highly prevalent in patients with cardiovascular diseases [11] and in particular heart failure (HF) and is an important predictor of adverse clinical outcomes [6]. Whilst oral iron supplements are available, these are poorly absorbed, leading to incomplete iron repletion, and have often significant gastrointestinal side effects, thereby limiting their usefulness. This is particularly important in the heart failure population as iron absorption may be further decreased and compliance reduced due to pill burden [12]. However, intravenous iron replacement has been shown to improve prognosis including quality of life, exercise capacity and hospitalizations for HF [6,7,13,14,15]. As a consequence, international guidelines for HF suggest screening patients for ID, with or without anemia, and to consider intravenous iron replacement in patients who are found to be deficient [16,17]. However, the recommendations differ in their strength. Whilst the European guidelines recommend that iron replacement should be considered to improve symptoms, exercise capacity and quality of life (QOL) and may be considered to improve hospitalizations, the AHA/ACC/HFSA guidelines only recommend that it is reasonable to use intravenous iron replacement to improve functional status and QOL. Thus, solidifying the available evidence on the effectiveness of intravenous iron replacement may lead to stronger recommendations for iron replacement in the heart failure population.

The underlying mechanisms of how ID affects cardiovascular function are still being elucidated. Recent studies have elegantly demonstrated a potential role for mitochondrial dysfunction to help explain adverse outcomes in patients with HF and co-existing ID. Through use of magnetic resonance spectroscopy (MRS) it has been shown that energetic stores in both skeletal as well as heart muscle are reduced in patients with ID [18,19]. This is important as the effects of iron replacement on quality of life and exercise capacity may be caused by improvement in either improved skeletal function, heart muscle function, or both. However, the beneficial effects of iron replacement on cardiovascular mortality are not well established. In this systematic review and meta-analysis, we assessed the effects of intravenous iron replacement on cardiovascular mortality, all-cause mortality as well as hospitalizations in patients with HF following completion of IRONMAN, the largest randomized controlled trials (RCT) in this field [15].

## 2. Materials and Methods

The systematic review and meta-analysis were conducted according to the Preferred Reporting Items for Systematic Reviews and Meta-analyses (PRISMA) guidelines [20] and was prospectively registered with PROSPERO (CRD42023384715). 

### 2.1. Type of Studies, Outcomes and Inclusion and Exclusion Criteria

We planned to only include RCTs comparing intravenous iron replacement to placebo or no treatment, in patients with heart failure and co-existing ID. We included studies reported in full-text or abstract form if sufficient data were available. We included all participants with a diagnosis of HF irrespective of etiology who were also diagnosed as iron deficient by any classification. 

The primary endpoint was cardiovascular mortality, secondary endpoints were all-cause mortality, hospitalizations for HF, and the combined endpoint of cardiovascular mortality and admission for HF. Studies had to report on either cardiovascular mortality or all-cause mortality to be included in this review. We did not examine studies that did not include a placebo or no treatment arm.

### 2.2. Search Methods 

We searched PubMed and Embase from inception until December 2022 without language restrictions or filters. The following search term was used: ((ferric derisomaltose OR ferric carboxymaltose OR iron sucrose OR iron dextran) AND heart failure AND Random*). Reference lists of key studies included in the systematic review were screened for additional references.

### 2.3. Data Collection and Analysis

After removal of duplicates, three reviewers (J.R., N.C. and V.B.) independently screened titles and abstracts. Full text articles were assessed for inclusion in the meta-analysis based on the presence of cardiovascular or all-cause mortality and based on the inclusion and exclusion criteria above by two independent reviewers (J.R. and V.B.). Any disagreements were resolved through discussion or if this was not possible by a third review author (V.S.V.). Covidence was used to document the selection process and to allow completion of a PRISMA flow diagram. A PRISMA checklist (Supplementary Materials) has been completed (Supplemental Material). 

### 2.4. Data Extraction and Management

Two authors (J.R. and N.C.) extracted the following data from the included studies using a standardized data collection sheet: First author and study name, year published, countries enrolling, number of patients, blinding details, main inclusion criteria, baseline characteristics of patients, definition of ID used, iron preparation used for treatment, follow up duration as well as primary and secondary endpoints. 

### 2.5. Risk of Bias Assessment 

Risk of bias in all included studies was assessed using Covidence, through ranking of seven domains as ‘low risk’, ‘high risk’ or ‘intermediate’ by two independent reviewers (NC and GM). We planned to assess publication bias visually and by funnel plot if at least ten studies reported on any outcome measure.

### 2.6. Data Synthesis and Quantification

For all outcomes, we collected data and reported odds ratios (ORs) with 95% confidence intervals. Meta-analysis was performed when at least two studies were identified that reported on the same outcome measure. Heterogeneity was assessed both qualitatively through comparing characteristics of the included studies and through the I2 statistic in each analysis with statistical significance defined as *p* < 0.05. We planned to analyze data using fixed effects model if heterogeneity was low or otherwise using random effects models in Review Manager 5.3.

### 2.7. Subgroup Analysis and Sensitivity Analysis

If possible, we planned to carry out the following pre-determined subgroup analyses: HFpEF vs. HFrEF, anemic vs. non-anemic patients, male vs. female and according to NYHA classification, HF etiology and Age. If enough data were available, we planned to perform a sensitivity analysis excluding studies with two or more domains of high risk of bias. Additionally, we planned to perform a ‘leave-one-out’ meta-analysis.

## 3. Results

### 3.1. Search Strategy

**Figure 1:**
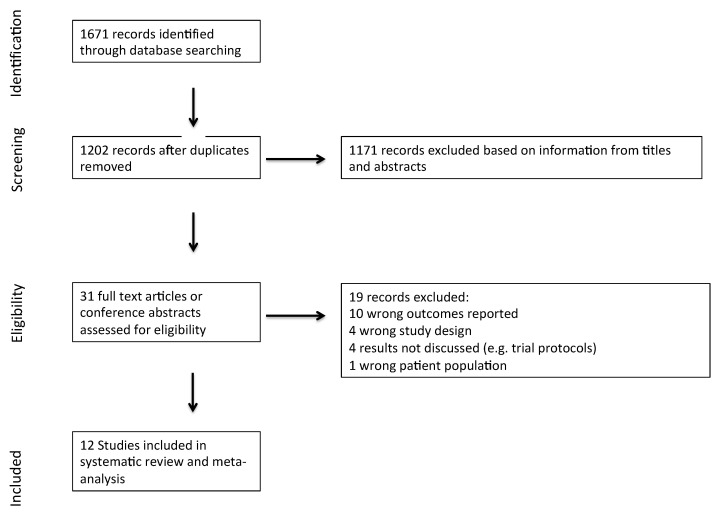
Preferred reporting items for systematic reviews and meta-analyses (PRISMA) flow diagram.

Our search yielded 1671 results (see Figure 1, PRISMA flow chart). After the removal of 469 duplicates, we screened titles and abstracts of 1202 records. Of these, 31 studies were identified for full-text review and 12 studies [6,7,8,15,21,22,23,24,25,26,27,28] were included in this review. Studies were excluded primarily as they did not report on cardiovascular mortality or all-cause mortality.

### 3.2. Characteristics of Included Studies and Risk of Bias Assessment

**Figure 2:**
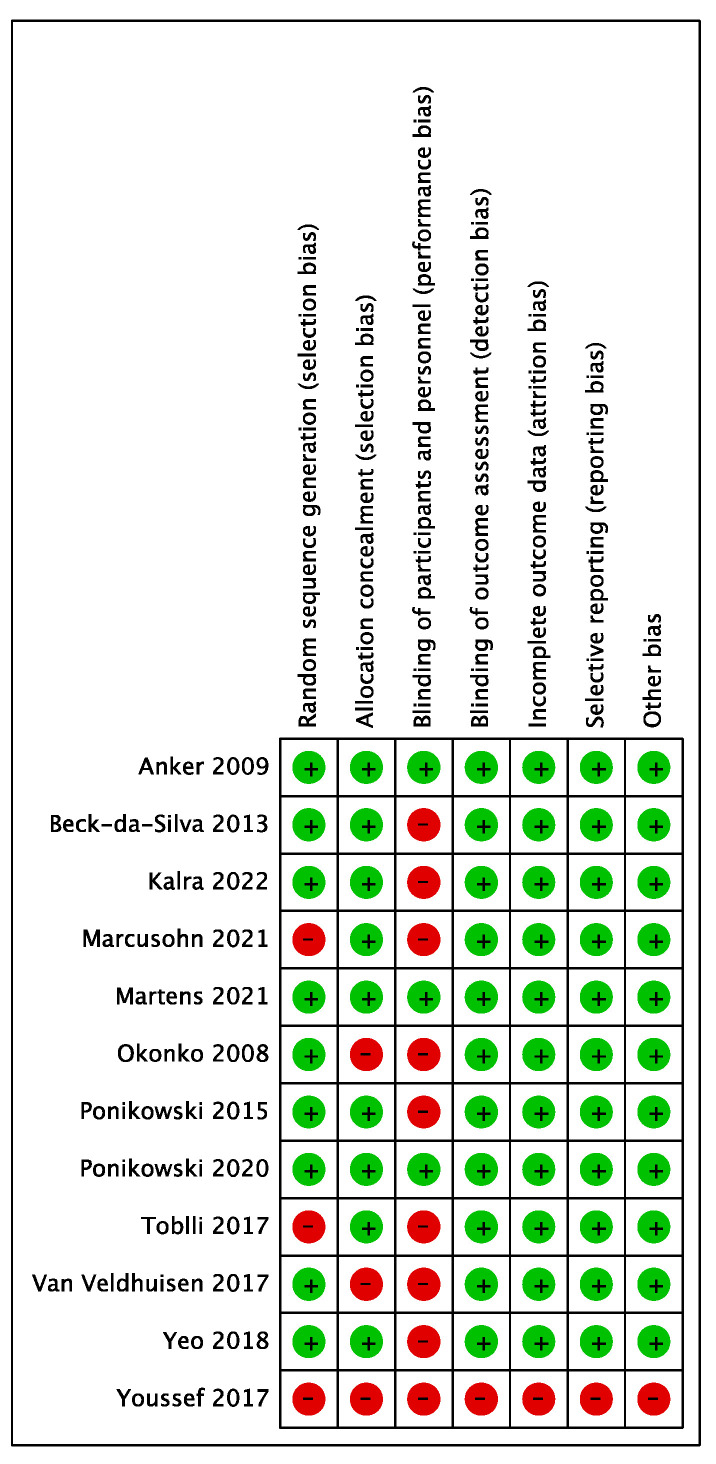
Risk of bias assessment. Red (-) indicates high risk and green (+) low risk in the studies included [6,7,8,15,21,22,23,24,25,26,27,28].

Characteristics of all included studies are summarized in Table 1 and the corresponding risk of bias assessment in Figure 2. The studies were heterogeneous in terms of study size and methodological quality. Importantly, the event rate of cardiovascular mortality or all-cause mortality was low, particularly in smaller pilot studies.

### 3.3. Primary Endpoint—Cardiovascular Mortality

**Figure 3:**
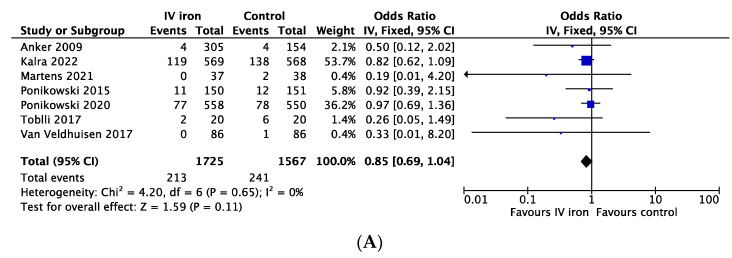

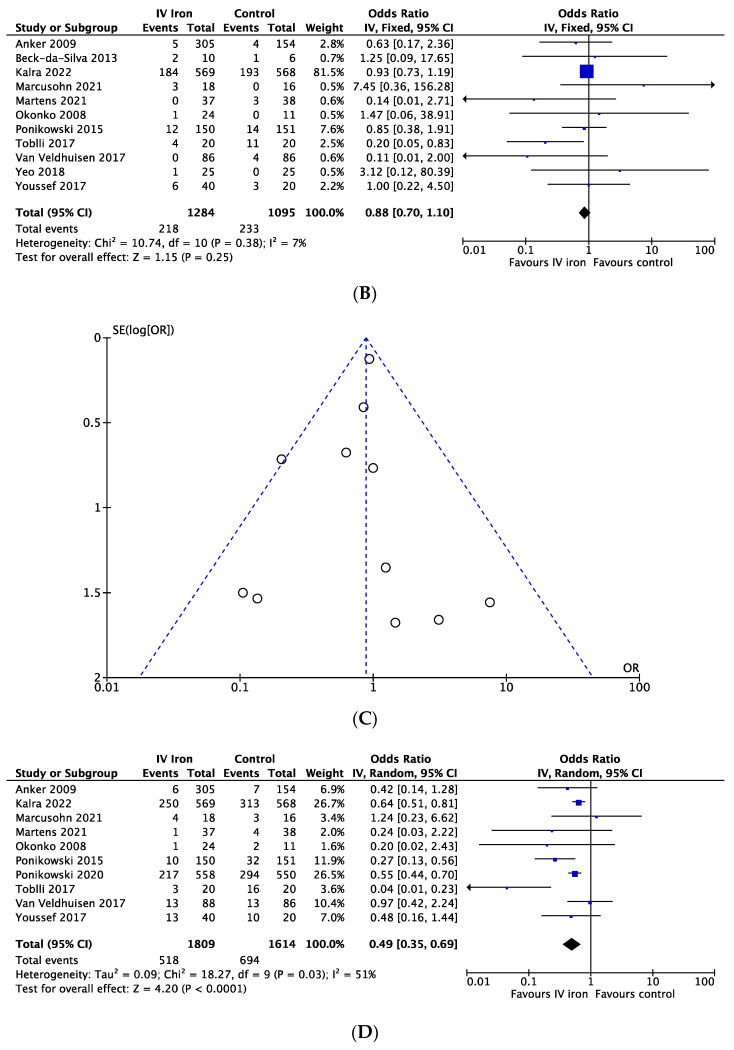

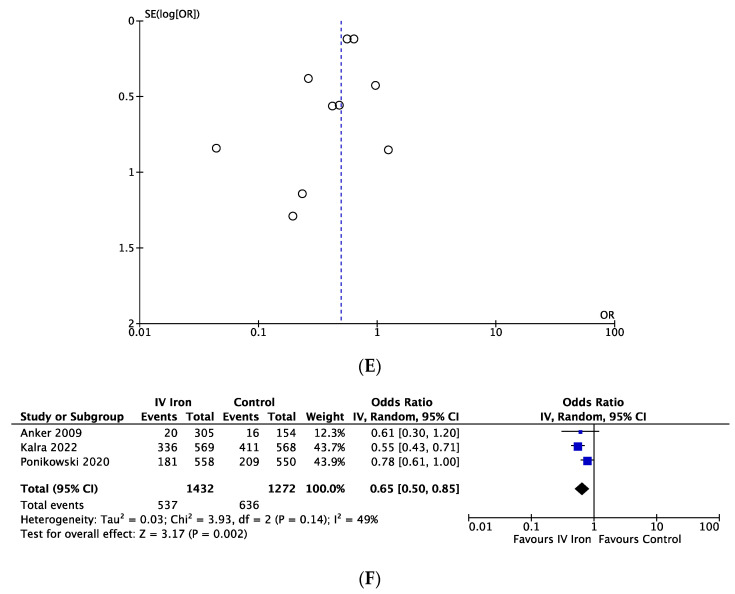
(**A**) Forrest plot for cardiovascular mortality with intravenous iron replacement compared to control using fixed effects model. (**B**) Forrest plot for all-cause mortality comparing both arms using fixed effects model. (**C**) Funnel plot for all-cause mortality. (**D**) Forrest plot for hospitalizations for heart failure with intravenous iron replacement compared to control using random effects model. (**E**) Funnel plot for hospitalizations for heart failure. (**F**) Forrest plot for hospitalizations for heart failure and cardiovascular death using random effects model [6,7,8,15,21,22,23,24,25,26,27,28].

Seven studies including 3292 patients reported on cardiovascular mortality or all-cause mortality where cardiovascular mortality could be adjudicated from the information provided in the full text articles [6,7,8,15,21,22,26]. Using a fixed effects model, 213 events/1725 patients in the intravenous iron replacement group and 241 events/1567 patients in the control group yielded an odds ratio of 0.85 (95% CI 0.69 to 1.04) (Figure 3A). No individual study reached statistical significance but, numerically, all studies included here reported odds ratios < 1. Funnel plot analysis was not performed as less than ten studies reported on the primary outcome.

### 3.4. Secondary Endpoints

#### 3.4.1. All-Cause Mortality

A total of 11 studies incorporating 2379 patients reported on all-cause mortality [5,6,7,8,15,21,23,24,25,26,28]. Using a fixed effects model, our meta-analysis estimates an odd ratio of 0.88 (95% CI 0.70 to 1.10) based on 218 events/1284 patients in the interventional arm and 233 events/1095 patients in the control arm (Figure 3B). Similarly to cardiovascular mortality, event rates for all-cause mortality were modest or small, except for the IRONMAN study [15], which included 1869 patients with a median follow up of 2.7 years. A funnel plot was not suggestive of significant publication bias (Figure 3C).

#### 3.4.2. Heart Failure Hospitalizations

We included ten studies (3423 patients) reporting on hospitalizations for heart failure [6,7,8,15,21,22,24,25,26,27]. Our random effects model estimates an odds ratio of 0.49 (95% CI 0.35 to 0.69) favoring intravenous iron replacement (Figure 3D). A funnel plot was not suggestive of publication bias (Figure 3E). Our findings were robust in a sensitivity analysis including only studies with one or less high-risk domains identified during risk of bias assessment. Similarly, this result was not altered in a leave-one-out sensitivity analysis.

#### 3.4.3. Heart Failure Hospitalizations and Cardiovascular Death

The pre-specified endpoint of cardiovascular death and hospitalizations for heart failure was reported only in three studies [6,15,22]. Of note, the three included studies were the largest in the field including 2704 patients. We estimate an odds ratio of 0.65 (95% CI 0.5 to 0.85) using a random effects model (Figure 3F). Due to the small number of studies included in this analysis we were unable to perform sensitivity analysis of funnel plot analysis.

#### 3.4.4. Endpoints in Chronic Heart Failure and Long Follow Up

Because mortality after an acute admission with heart failure may be higher, we also conducted additional subgroup analyses including only studies with chronic heart failure as opposed to acute heart failure and in a separate analysis only studies with a long follow up of one year or greater. Including chronic heart failure studies only, the overall results were not altered (see Supplemental Material). Similarly, including only studies with a long follow up did not alter the main results of this study (see Supplemental Material).

## 4. Discussion

To our knowledge, this systematic review and meta-analysis is the first to report on outcomes of intravenous iron replacement therapy in patients with HF and co-existing ID since the completion of the IRONMAN trial [15]. In the present analysis, we focused on hard clinical outcomes and in particular our primary outcome cardiovascular mortality but also all-cause mortality, hospitalizations for heart failure and a combination of hospitalizations and cardiovascular mortality. Our findings suggest a lack of sufficient evidence to conclude whether intravenous iron replacement decreases cardiovascular mortality in patients with HF and ID. All studies included in this systematic review and meta-analysis numerically reported lower cardiovascular event rates in the intravenous iron replacement groups compared to placebo or standard of care; however, the differences are not statistically significant in individual studies or the present meta-analysis. Similarly, there was not enough evidence to conclude on any effect on all-cause mortality with intravenous iron replacement.

Hospitalization for heart failure and the combined endpoint of hospitalizations for heart failure and cardiovascular death were significantly reduced in the intravenous iron replacement group. These secondary endpoints are clinically important for patients, especially their quality of life, and importantly for costs incurred in health care systems. Whilst our study did not demonstrate improved cardiovascular or all-cause mortality with intravenous iron replacement, the benefits we see with hospitalizations have important implications. Overall, our findings suggest that international heart failure guidelines [16,17] could recommend intravenous iron replacement not only to improve functional outcomes but also to reduce hospitalizations.

The underlying mechanisms of how ID and, in turn, IV iron supplementation affect cardiovascular outcomes remain incompletely understood. On a cellular level, ID induces reduced mitochondrial electron transfer chain activity, reduced mitochondrial respiration, a switch towards immature glycolytic metabolism, reduced cellular ATP availability and reduced contractility of human cardiomyocytes [29,30]. Importantly, at least some of the changes occurring during ID are reversible upon replenishment of intracellular iron. Similar effects appear to occur in vivo in human hearts and skeletal muscle as assessed by magnetic resonance spectroscopy [18,19]. These studies have elegantly demonstrated that ID is associated with reduced cardiac energetic stores such as the phosphocreatine (PCr) to ATP ratio. IV iron replacement, in turn, improves phosphocreatine recovery half-time, a measure of energetic stores, at least in skeletal muscle. The effects of IV iron replacement on exercise capacity in patients with heart failure and ID may, therefore, be mediated in part through effects on skeletal and cardiac muscle.

The current study is not without limitations. We used aggregate data from published reports of RCTs to conduct this meta-analysis. Many of the included studies were small in size and the results of this systematic review and meta-analysis were mainly driven by the findings of the largest four trials [6,7,15,22]. Individual patient data meta-analysis is sometimes considered the ‘gold standard’ of systematic reviews and it would be desirable to perform such an analysis on these four trials; however, this is time-consuming and requires collaboration between various research teams [31]. 

The included studies also differed in the HF population under study including whether patients had acute or chronic heart failure. For example, by far the largest studies included in this analysis are the AFFIRM-AHF [22] and the IRONMAN [15] studies. The former study recruited patients during an admission for acute heart failure whilst the latter recruited patients with stable chronic HF. Nevertheless, at least for HF hospitalizations, both of these studies support the use of intravenous iron replacement suggesting that this therapy is beneficial across a wide spectrum of HF populations and presentations. 

Studies included in this review used various intravenous iron preparations including ferrous carboxymaltose (FCM), ferric derisomaltose (FDM), iron sucrose (IS) and sodium ferric gluconate complex (SFGC) (Table 1); however, to our knowledge, there are no head-to-head comparisons of these preparations available at present. It is therefore unknown, whether any of the preparations are more effective than the others. Most data in the current systematic review and meta-analysis stems from RCTs using FCM or FDM in the treatment arms. Prior to the IRONMAN trial, all larger RCTs had used FCM as the intravenous iron preparation. The additional evidence from the IRONMAN suggests that there may well be a class effect of intravenous iron replacement. However, larger trials of the other preparations (IS and SFGC) would be needed to investigate whether they are equally effective.

We were unable to perform subgroup analysis of different patient groups such as HFpEF and HFrEF, various NYHA severity, HF etiology, patients age, renal function or the presence of absence of anemia as this data could not be extracted in this aggregate meta-analysis. Such an analysis would be desirable in the setting of an individual patient data meta-analysis in the future. For example, using individual patient data, a direct comparison of patients with NYHA severity II and III could be performed, helping to clarify which patients are more likely to benefit. Similarly, comparisons could be made in different HF groups such as HFpEF and HFrEF, according to HF etiology, or other factors such as the time interval from initial diagnosis of heart failure. 

Many of the trials included in here relied on the current ‘guideline definition’ [16] for identification of patients with ID, i.e., ferritin < 100 µg/L or ferritin 100–299 µg/L and transferrin saturation (TSAT) < 20% used in pivotal early trials in the field [6,7]. However, we are becoming increasingly aware that a different definition of ID based on either TSAT measurement or serum iron concentrations rather than ferritin might be more appropriate [9,10]. The rationale for the use of alternative definitions is that ferritin can be released into the circulation during any sort of cell damage and inflammatory processes and indeed have been found to be elevated in patients diagnosed as iron deficient through the gold-standard of bone marrow biopsy [9]. Furthermore, since traditional markers of ID may not always correlate with tissue iron content, cardiac MRI, utilizing specialized techniques like T2*, has been proposed as a technique to assess accurately myocardial iron content [32,33]. Identifying patient populations who are most likely to benefit from intravenous iron replacement could, in theory, improve the efficacy of this therapeutic approach.

## 5. Conclusions

This is the largest systematic review and meta-analysis of intravenous iron therapy in patients with HF and co-existing ID to date, and the only one to include the largest trial, IRONMAN [15], in this field. We show that whilst there is a move towards reduced cardiovascular and all-cause mortality in the intravenous iron group, these did not reach statistical significance. However, hospitalization rates for HF as well as the combined endpoint of cardiovascular mortality and HF hospitalizations were significantly reduced in the intravenous iron therapy groups. Our findings are important, as they confirm the appropriate use of intravenous iron in a wide range of patients with HF. However, at the same time, future studies are desirable to identify the phenotype of patients most likely to benefit, as we were unable to make such conclusions based on the present study and the RCTs included in it. Furthermore, it will be important to clarify, in larger studies, whether cardiovascular mortality might also be reduced.

## Figures and Tables

**Table 1:** Characteristics of included studies.

Author/Year	Study Name	Countries	Number of Patients	Blinding	Definition of ID	IV Iron Form	Main Inclusion Criteria	Age (years)	Female (%)	LVEF (%)	Ischemic Cardiomyopathy (%)	NYHA III-IV (%)	eGFR (ml/min/1.73m^2^)	Follow Up Duration (weeks)
Anker 2009 [6]	FAIR-HF	11 countries (Europe and Argentina)	459	Double	Guideline definition	FCM	Ambulatory NYHA II-III LVEF < 45% Hb 95–135g/L + ID	67.8 (10.3) (FCM) 67.4 (11.1) (placebo)	159 (52.3) (FCM) 85 (54.4) (placebo)	31.9 (5.5) (FCM) 33 (6.1) (placebo)	245 (80.6) (FCM) 123 (79.4) (placebo)	251 (82.6) (FCM) 126 (81.3) (placebo) (NHA IV excl)	63.8 (21.2) (FCM) 64.8 (25.3) (placebo)	26
Beck-da-Silva 2013 [28]	IRON-HF	Brazil	23	Double	Ferritin < 500 µg/L AND TSAT < 20%	IS	LVEF < 40% NYHA II-IV Able to perform ergospirometry	66.9 (8.3) (IS) 68.9 (10.1) (placebo)	30.4	25.2 (8.6) (IS) 30.7 (7.4) (placebo)	39.1%	NA	NA	12
Kalra 2022 [15]	IRONMAN	UK	1137	Single (masked outcomes). Open label administration	Ferritin < 100 or TSAT < 20%	FDM	LVEF < 45% Iron deficient Symptomatic HF	73·2 (66·7–80·1) (FDM) 73·5 (67·1–79·1) (usual care)	142 (25%) (FDM) 158 (28%) (usual care)	32 (25–37) (FDM) 35 (26–38) (usual care)	331 (58%) (FDM) 316 (56%) (usual care)	241 (42) (FDM) 248 (44) (usual care)	51·7 (38·1–68·1) (FDM) 50·1 (37·8–68·6) (usual care)	117
Marcusohn 2021 [25]		Israel	34	Single	Guideline definition	SFGC	Acute admission with: -Hb 8–14 -ID -NT-proBNP > 300 pg/mL -IV loop diuretics	71.5 (66.0 to 78.0)	11 (32.4)	35 (20 to 55)	N/A	N/A	N/A	24
Martens 2021 [21]	IRON-CRT	Belgium	75	Double	Guideline definition	FCM	NYHA II-IV CRT > 6 months with ≥98% biventricular pacing LVEF ≤ 45% ID	72 (12) (FCM) 73 (9) (placebo)	11 (30) (FCM) 13 (34) (placebo)	33 (8) (FCM) 34 (7) (placebo)	19 (51) (FCM) 24 (63) (placebo)	15 (41) (FCM)	56 (25) (FCM) 51 (22) (placebo)	13
Okonko 2008 [24]	FERRIC-HF	Poland, UK	35	No	Guideline definition	IS	NYHA II-III LVEF ≤ 45% pVO_2_/kg ≤ 18 mL/kg/min ID	64 (14) (IS) 62 (11) (control)	7 (29) (IS) 3 (27) (control)	N/A	18 (75) (IS) 8 (73) (control)	11 (46) (IS) 5 (45) (control)	N/A	18
Ponikowski 2015 [7]	CONFIRM-HF	Austria, Italy, Poland, Portugal, Russia, Spain, Sweden, UK, and Ukraine	304	Double	Guideline definition	FCM	NYHA II-III LVEF ≤ 45% ID and Hb < 15 g/dl Able to complete 6 min walking test	68.8 (9.5) (FCM) 69.5 (9.3) (placebo)	67(45) FCM 74 (49) (placebo)	37.1 (7.5) (FCM) 36.5 (7.3) (placebo)	125 (83) (FCM) 126 (83) (placebo)	70 (47) (FCM) 60 (40) (placebo)	66.4 (21.7) (FCM) 63.5 (20.9) (placebo)	52
Ponikowski 2020 [22]	AFFIRM-AHF	121 sites in Europe, South America, and Singapore	1132	Double	Guideline definition	FCM	ADHF LVEF ≤ 50%	71.2 (10.8) (FCM) 70.9 (11.1) (placebo)	244 (44%) (FCM) 250 (45%) (placebo)	32.6 (9.6) (FCM) 32.7 (10.0) (placebo)	265 (47%) (FCM) 257 (47%) (placebo)	286 (51) (FCM) 299 (54) (placebo)	<60: 292 (52%) (FCM) 288 (52%) (placebo)	52
Toblli 2017 [26]	N/A	Argentina	40	Single	serum ferritin < 100 µg/L and/or TSAT ≤ 20%	IS	NYHA II-IV LVEF ≤ 35% ID and anemia	76 (7) (IS) 74 (8) (saline)	7 (35%) (IS) 8 (40%) (saline)	31.3 (3.7) (IS) 30.8 (1.7) (saline)	?	2.9 (0.7) (IS) 2.9 (0.6) (saline)	?	260
Van Veldhuisen 2017 [8]	EFFECT-HF	Australia, Belgium, France, Germany, Italy, The Netherlands, Poland, Russia, and Spain	174	No	Guideline definition	FCM	NYHA II-III. Optimized HF medications, LVEF ≤ 45%, ID	63 (12) (FCM) 64 (11) (standard care)	26 (30%) (FCM) 17 (20%) (standard care)	33 (9) (FCM) 31 (8) (standard care)	?	25 (29) (FCM) 32 (37) (standard care)	52 (13) (FCM) 51 (12) (standard care)	24
Yeo 2018 [23]	PRACTICE-ASIA-HF	Singapore	50	Single	ferritin < 300 µg/L and TSAT < 20%	FCM	ADHF, anemia and ID, able to do 6 min walk test, age > 21 years	61.1 (10.8) (FCM) 64 (10) (Saline)	6 (25%) (FCM), 5 (20%) (Saline)	38.8 (17.5) 33.2 (14.8)	?	?	?	12
Youssef 2017 [27]	N/A	Egypt	60	Single	Guideline definition	IS	ADHF NYHA III-IV, LVEF < 40, Anemia and ID	?	?	<40%	?	100%	?	13

Note: Guideline definition of iron deficiency: ferritin < 100 µg/L or ferritin 100–299 µg/L and transferrin saturation (TSAT) < 20%. Data are mean (SD), n (%), or median (IQR). Abbreviations: ADHF acute decompensated heart failure; CRT cardiac resynchronization therapy; FCM ferric carboxymaltose; FDM ferric derisomaltose; ID iron deficiency; IS iron sucrose; HF heart failure; NYHA New York Heart Association; LVEF left ventricular ejection fraction; SFGC sodium ferric gluconate complex.

## Data Availability

All relevant data generated in this study are available in this article.

## Supplementary Materials

The following supporting information can be downloaded at: https://www.mdpi.com/article/10.3390/jcdd10030116/s1, Table S1: PRISMA checklist. Ref. [34] mentioned in the supplementary material.
